# Evaluation of terminal room cleaning and disinfection after patients infected/colonized with vancomycin-resistant enterococci (VRE) discharge in an adult intensive care unit of a medical center

**DOI:** 10.1186/2047-2994-4-S1-P202

**Published:** 2015-06-16

**Authors:** SW Wu, Y-T Jao, C-T Hung, P-W Yang, T-H Hung, S-H Lin, CH Huang, C-Y Lin, Y-H Chen

**Affiliations:** 1 Department of Infection Control, Department of Internal Medicine, Kaohsiung Medical University Hospital, Kaohsiung Medical University, Kaohsiung, Taiwan, Province of China, Kaohsiung, Taiwan, Province of China; 2 Division of Infectious Diseases, Department of Internal Medicine, Kaohsiung Medical University Hospital, Kaohsiung Medical University, Kaohsiung, Taiwan, Province of China, Kaohsiung, Taiwan, Province of China

## Introduction

Contaminated environment is a risk factor of hospital-acquired infection. Inappropriate cleaning/disinfection of environment may enhance vancomycin-resistant enterococci (VRE) transmission from environments to patients.

## Objectives

The investigation was made after 3 patients with VRE infection/colonization noted in November 2014 in an adult intensive care unit of a medical center in Taiwan.

## Methods

Samples were taken from frequently contacted surfaces including bed rails, bed control, monitor control panel, EKG touch panel, work table and drawer handles in rooms previously occupied by VRE infected/colonized patient. The ATP (Adenosine Tri-Phosphate) bioluminescence (normal reference: < 250 Relative Light Unit (RLU)) and swab culture was performed before and after cleaning and disinfection of environment on 6 checkpoints each room. Totally, 36 samples were taken to evaluate the effectiveness of cleaning and disinfection. The swab cultures were also performed on hands of healthcare workers.

## Results

Totally 70 samples were taken including 56 samples from environments and 14 samples from hands of healthcare workers. Before cleaning and disinfection, all 18 samples from environments were more than 250 RLU by ATP bioluminescence. The non-qualified rate was 100%. After cleaning and disinfection, 8 out of 18 (44%) check points were detected abnormal by ATP bioluminescence. These 8 check points included 3 samples from bed rails (544~4869RLU), 2 samples from bed controls (1800~3231RLU) and each one sample from drawer handle, EKG touch panel and IV pump control panel (375~1607RLU).

## Conclusion

VRE can survive for months on environments and patients. Hands of healthcare worker may transmit VRE and cross-infection between patients may occur. Through emphasizing the importance of environmental cleaning/disinfection and taking appropriate infection control measures, hospital-acquired infection can be prevented. Hand hygiene and environmental cleaning/disinfection are essential to ensure patient safety.

## Disclosure of interest

None declared.

